# The Neuropharmacology of (-)-Stepholidine and its Potential Applications

**DOI:** 10.2174/157015907782793649

**Published:** 2007-12

**Authors:** Kechun Yang, Guozhang Jin, Jie Wu

**Affiliations:** 1 Division of Neurology, Barrow Neurological Institute, St. Joseph’s Hospital and Medical Center, Phoenix, AZ 85013, USA; 2 Department of Pharmacology, State Key Laboratory of Drug Research, Shanghai Institute of Materia Medica, Shanghai Institute of Biological Sciences, Chinese Academy of Sciences, Shanghai, China

**Keywords:** Stepholidine, dopamine D1 receptor, dopamine D2 receptor, schizophrenia, Parkinson's disease.

## Abstract

(-)-Stepholidine (SPD), a natural product isolated from the Chinese herb *Stephania*, possesses dopamine (DA) D1 partial agonistic and D2 antagonistic properties in the nigrostriatal and mesocorticolimbic DAergic pathways. These unique dual effects have suggested that SPD can effectively restore previously imbalanced functional linkage between D1 and D2 receptors under schizophrenic conditions, in which, SPD improves both the negative and positive symptoms of schizophrenia. SPD also relieves the motor symptoms of Parkinson’s disease (PD) when co-administered with Levodopa. Furthermore, SPD exhibits neuroprotective effects through an antioxidative mechanism and slows down the progression of neuronal degeneration in the substantia nigra (SN) of PD patients and/or animal models. Therefore, SPD is a novel, natural compound with potentially therapeutic roles in the treatment of schizophrenia and/or PD.

## 1. INTRODUCTION

**Fig. (1).**
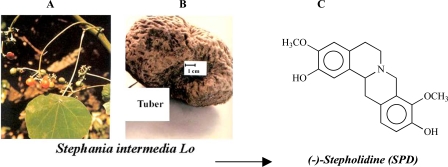


(-)-Stepholidine (SPD), extracted from the Chinese medicinal plant *Stephania intermedia Lo* (Fig. **1**), belongs to an alkaloid of tetrahydroprotoberberines (THPBs). Initially, SPD was reported to decrease blood pressure without exerting any adverse effect on the heart [75, 84], and exhibited analgesia and sedative effects on the central nervous system (CNS) [6, 75]. Thereafter, the most important findings, from behavioral, electrophysiological, biochemical, immunohistochemical and pharmacological studies, have revealed that SPD is a dopamine (DA) D1 receptor partial agonist and D2 receptor antagonist [10, 11, 20, 28, 34, 37, 60, 62, 66, 82, 83, 87]. This unique neuropharmacological feature appears to have therapeutic potential because based on the concept of schizophrenia pathogenesis, an imbalance of brain DA function arises from dysfunction of D1 receptors in the medial prefrontal cortex (mPFC) and hyperactivity of D2 receptors in lower structures of cortex, such as the ventral tegmental area (VTA) and nucleus accumbens (NAc). The hypothesis of DA receptor imbalance has resulted in the development of antipsychotic medicine. SPD is a novel, natural compound, and may serve as a potential candidate for the treatment of schizophrenia and/or PD. This review focuses on the recent advances of SPD neuropharmacology.

## 2. DAERGIC PATHWAYS AND RECEPTORS IN THE CNS

The DAergic system in the CNS plays important roles in the control of cognitive, motivational, motor and endocrine functions. DA exerts its pharmacological effects *via* specific DAergic receptors, which are particularly expressed in the nigrostriatal and mesocorticolimbic pathways.

The nigrostriatal pathway, which is comprised of DA-synthesizing neurons of the substantia nigra pars compacta (SNc) that project to the striatum, contains nearly 80% of all the DA in the CNS. This pathway is crucial for motor coordination, and the degeneration or dysfunction of this pathway results in the characteristics of Parkinson’s disease (PD). The mesocorticolimbic pathway, which arises in the VTA and projects to the PFC and limbic areas, such as the NAc and the olfactory tubercle, is involved in regulating motivational behavior, learning, memory and cognition. The degeneration or dysfunction of this DAergic pathway is implicated in the development of schizophrenia. Therefore, this pathway may be the target of antipsychotics.

Recently, five subtypes of DAergic receptors, which belong to the family of seven-transmembrane heterotrimeric G-protein coupled receptors, have been cloned and identified. Two different categories of DAergic receptors, termed D1-like (D1 and D5) and D2-like (D2-D4), have been identified on the basis of DNA sequence similarities [61]. D1 receptors interact with G_s_ proteins to positively activate adenylyl cyclase, while D2 receptors act through G_i_ and G_o_ proteins to inhibit cAMP production. Thus, D1 and D2 receptors exert opposing effects and balance the two signaling pathways through the activation and inhibition of adenylyl cyclase activity. Although D1 and D2 receptors belong to distinct subfamilies of DA receptors, several lines of evidence indicate there is an important functional linkage between these two subclasses of receptors.

Behavioral, biochemical and electrophysiological evidence has revealed synergism between D1 and D2 receptors [17, 21, 44, 65]. Calabresi *et al*. demonstrated that the abolished long-term depression (LTD) of synaptic transmission in the striatum after DA depletion could be restored by exogenous DA or co-administration of specific D1 and D2 agonists, but not by application of either selective agonist alone [8]. Therefore, co-activation of D1 and D2 receptors is required for LTD in the striatum. Using confocal microscopy, Aizman *et al*. observed that virtually all striatal neurons contained both D1 and D2 receptors  [3]. Using both immunoprecipitation and immunohistochemistry methods, Lee *et al*. demonstrated that D1 and D2 receptors are co-expressed and co-localized within striatal neurons and that these two receptors are part of the same heteromeric protein complex and a phospholipase C-mediated calcium pathway that is only activated by both D1 and D2 receptor co-activation [47]. These results demonstrate that D1 and D2 receptors are co-expressed in neurons of rat striatum, suggesting the possibility that there may be a direct D1-D2 receptor association.

Therefore, functional synergism between D1 and D2 receptors is necessary for maintaining some normal physiological functions, and once the functional linkage between the two receptors is broken, disorders, such as schizophrenia, may develop.

## 3. DUAL EFFECTS OF SPD ON THE NIGROSTRIATAL PATHWAY 

The DAergic system in the SNc, substantia nigra pars reticulata (SNr), neostriatum, globus pallidus, and subthalamic nucleus is known to play a pivotal role in the control and coordination of movement. In 1968, Ungerstedt developed a new technique for lesioning the nigrostriatal DA system by injecting 6-hydroxy-DA (6-OHDA) into the SN, which produced unilateral anterograde degeneration of the nigro-neostriatal DA system and constituted the first rat hemiparkinsonian model [67]. Using this model, subsequent study showed that rotational behavior could be induced by systemic administration of a DA agonist or amphetamine. It is well-known that amphetamine can increase DA turnover mainly by releasing DA from nerve terminals, thus seriously worsening the existing imbalance in DA neuronal activity between the lesioned and contralateral sides. Collectively, these effects result in the animal rotating towards the lesioned side [68].

In the 1980s, Jin and colleagues started examining the pharmacological effects of SPD and its analogs using the 6-OHDA-induced PD rat model [60]. They first reported that SPD produced a significant contralateral rotation, which was mimicked by the D1 agonist SKF38393 and blocked by the D1 antagonist SCH23390 [29, 31, 34, 60], suggesting that SPD exhibits a D1 agonistic effect. Subsequent study showed that SPD exhibited a direct agonist effect on neurons in the nigrostriatum and SNr [82]. Moreover, *in vitro* radioligand binding assay demonstrated that SPD was able to bind to the D1 receptor at two sites, a high-affinity site (K_i _= 3.9 nM) and a low-affinity site (K_i _= 126 nM), using calf striatal membrane preparations [16]. These results strongly suggest that SPD exerts an agonistic effect on D1 receptors under 6-OHDA-induced lesioned conditions.

In contrast, in control and reserpinized rats (rodents treated with reserpine at 1 mg/kg for 6 days to deplete endogenous DA), SPD exerts an antagonist effect on the D1 receptor. SPD reversed and/or significantly attenuated SNc DA cell firing induced by SKF38393 or by the mixed DA receptor agonist apomorphine (APO) in reserpinized rats. This effect was similar to that produced by SCH23390 [62, 64]. It has been shown that SPD only partially (51-53%) reverses the effects of SKF38393 but completely (100%) reverses the effects of SCH23390 [64]. Zou *et al*. demonstrated that SPD displays D1 agonistic effects at a low dose (0.5 or 1 mg/kg) but D1 antagonistic effects at a high dose (4 or 10 mg/kg) when co-administered with APO during behavioral experiments [90]. Furthermore, binding experiments showed that SPD binds to the D1 receptor with high affinity (K_i _= 13 nM, [76]), but exhibits much lower affinity for other receptors [35, 84].

What is the mechanism by which SPD exhibits this controversial effect on the D1 receptor? Although the exact reasons for this difference are still uncertain, it is clear that SPD shows D1 agonistic action in the presence of SCH23390 and exhibits antagonistic effects in the presence of SKF38393, meaning that as a partial agonist, SPD displays a large range of intrinsic activities at the same D1 receptors depending on different conditions. Another possible reason explaining this controversial effect is that the D1 receptor may exhibit different sensitivities under these two conditions. In the 6-OHDA-induced lesioned model, a relatively large amount of endogenous DA is depleted, which consequently enhances D1 receptor sensitivity. However, in normal rats, D1 receptors are not sensitive enough to observe the agonistic effects of SPD [62, 90].

In conclusion, SPD modulates D1 receptors depending on the receptor’s status. Under normal conditions, SPD works as a D1 receptor antagonist, while in the 6-OHDA-induced PD model, SPD serves as a partial agonist.

In addition to modulating D1 receptor function, SPD also show the ability to bind to the D2 receptor. Although its affinity for the D2 receptor (K_i _= 85 nM) is 4-7 times lower compared to the D1 receptor (K_i _= 13 nM) [76], SPD shows much lower or poor affinity for other receptors [35, 84]. It has been reported that SPD also modulates D2 receptor function. In normal rats, SPD antagonized APO-induced stereotypy [84], reduced the inhibitory effect of APO on the firing activity of nigral DA neurons and shifted the dose-response curve to the right [29], increased striatal Levodopa (L-dopa) and 3,4-dihydroxyphenylacetic acid accumulation induced by NSD1015 (a decarboxylase inhibitor) [26], and increased striatal DA release [30]. Moreover, SPD antagonized or reversed D2 receptor-mediated inhibition of synaptosomal adenylate cyclase (AC), which can be isolated from rat striatum, by affecting regulation of G_i_ proteins on D2 receptor-coupled AC [28]. In reserpinized rats, SPD reversed N0437 (a D2 receptor agonist)-induced inhibition of DA cell firing in the SNc, suggesting that SPD acts as a D2 receptor antagonist [62]. In 6-OHDA-induced lesioned rats, unlike its D1 receptor agonistic effect, SPD induced animal rotation towards the intact side by blocking D2 receptors [15, 34, 37, 86]. Therefore, SPD is a D2 antagonist.

## 4. DUAL EFFECTS OF SPD ON THE MESOCORTICOLIMBIC PATHWAY

SPD-induced dual D1 receptor agonistic and D2 receptor antagonistic effects have also been demonstrated in mesocorticolimbic structures, including the mPFC, NAc, and VTA DAergic systems. An *in vivo* electrophysiological study in rats demonstrated that at a low dose (1-64 μg/kg), SPD increased VTA DA cell firing and attenuated APO-induced inhibition of DA cell spontaneous firing, suggesting that SPD blocks D2 receptors located on VTA DA cells, thereby feed-back regulating spontaneous activity [63]. However, at a high dose (0.6-3.3 mg/kg), SPD continuously decreased the amplitude of spikes from spontaneously-firing cells until the majority (4/6) of VTA DA neurons became inactive. This manner of inhibition by SPD was reversed by APO and was different from inhibition resulting from APO-induced hyperpolarization. In addition, SPD was reported to produce a depolarization-induced inactivation (cessation of spontaneous firing due to excessive depolarization) of DA neurons in the VTA [37, 63].

Additional *in vivo* experiments using rats have demonstrated that intravenous (i.v.) administration of SPD (from 0.01 mg/kg to 2 mg/kg, each dosage doubled every 2 minutes) produced two opposing effects on the spontaneous firing of NAc neurons. Since D2 receptors on VTA DA neurons are 3-10-fold more sensitive to SPD than those in the NAc (which receives neuronal input from the VTA), a low dose (0.02-0.08 mg/kg) of SPD blocked D2 receptors on VTA DA neurons [87] and reduced DA release from nerve terminals in the NAc [89]. Therefore, a low dose of SPD produces an inhibitory effect on the spontaneous firing of NAc neurons due to this disinhibitory effect. However, a high dose (0.08-2 mg/kg) of SPD produces a biphasic effect (decrease followed by increase) on the firing activity of NAc neurons [87], meaning inhibitory then subsequently excitatory effects on the spontaneous firing of NAc neurons. The inhibitory effect is due to the previously mentioned disinhibitory effect *via* blockade of VTA D2 receptors. With pretreatment of spiperone (a D2 antagonist), only an excitatory effect is observed, but the excitatory effect is reversed by SCH23390, which suggests that SPD works as a D1 receptor agonist and induces excitation [87]. The D1 agonistic effect of SPD on spontaneous firing, however, is not observed in the NAc using direct microiontophoresis [88], suggesting that the D1 agonistic effect of SPD (i.v.) on NAc DA neurons is probably mediated by some indirect action in the mesocorticolimbic system. Actually, emerging evidence suggests that SPD-induced excitation is indirectly mediated *via* the mPFC D1 receptor [37, 88, 89]. Furthermore, an immunohistochemical study confirmed that D1 receptors are located on mPFC pyramidal neurons, which project directly to the NAc with glutamate-mediated (excitatory) efferents [5]. Therefore, it is possible that SPD directly activates D1 receptors on mPFC glutamatergic neurons that project to the NAc, thus indirectly influencing neuronal activity in the NAc. Consequently, the biphasic effect in the NAc induced by SPD is likely mediated by its antagonistic action on D2 receptors in the VTA and its agonistic effect on D1 receptors located in the PFC meso-accumbens DA system [87].

## 5. EFFECTS OF SPD ON PERIPHERAL DA RECEPTORS AND CA^2+^ CHANNELS AND ITS ANTIHYPERTENSIVE ACTION

It is well-known that specific DA receptors are expressed in both the CNS and extracerebral structures**. **In particular, DA receptors are present on vascular smooth muscle cells (vascular DA receptor) [79] and on postganglionic sympathethic nerve terminals (neuronal DA receptor) [32]. It has also been demonstrated that peripheral DA receptors are expressed in mammals at many sites, such as vascular smooth muscle, autonomic nerve endings in the cardiovascular system, autonomic ganglia, the kidneys, and so on [73]. Functionally, activation of the vascular DA receptor (D1 receptor) causes smooth muscle relaxation, whereas activation of the neuronal DA receptor (D2 receptor) reduces the response of the heart to nerve stimulation by depressing noradrenaline release [22, 27, 45, 48]. Thus, peripheral DA receptors participate in the regulation of peripheral vascular resistance, and in turn improve cardiovascular [33] and renal functions including vasodilatation, diuresis and natriuresis [12].

Evidence suggests that hypertension results from overactivity of central DA neurons in spontaneously hypertensive rats (SHRs). The prevailing opinion is that D2-like receptor function is upregulated in SHRs [43, 51, 71]. In addition to the central DA receptor system, renal and adrenal DAergic dysfunction may also play roles in the pathophysiology of hypertension. In human hypertension, a reduced ability of DA to regulate sodium excretion (most likely a D1-like receptor mechanism is impaired) has been reported [4, 39, 40]. Differences in D1-like receptor regulation of diuresis, natriuresis, Na^+^-K^+^-ATPase activity, and rat renal protein kinase C isoform expression between normotensive and hypertensive rats suggest that renal D1-like receptor dysfunction may be the mechanism responsible for generation of hypertensive rats [78]. Gordon *et al*. discovered that aberrant DAergic regulation of aldosterone secretion mediated *via* DA D2-like receptors may also contribute to hypertension [23]. The absence of interactions between D1/D2 receptors has also been reported in the kidneys of SHRs, but not in normotensive rats [70]. In addition, it has been suggested that stimulation of D1- or D2-like DAergic receptors may constitute a therapeutic strategy for the treatment of hypertension [4].

As mentioned above, SPD is a D1 receptor partial agonist and D2 receptor antagonist in the CNS, and studies have demonstrated that SPD also exerts dual effects on peripheral DA receptors. SPD (0.1-10 μM) shifts dose-response curves to the right and decreases the maximal response of both fenoldopam (a selective D1 agonist)-induced and propyl-butyl-DA (a selective D2 agonist)-induced vasorelaxation, showing a non-competitive antagonistic action on both D1 and D2 receptors. In addition, SPD (0.1-100 μM) induces slight but dose-dependent vasorelaxation of renal and pulmonary arteries, thereby displaying D1 agonistic activity [81].

It is possible that SPD may be a promising antihypertensive agent due to its interaction with both central and peripheral DA receptors. Furthermore, the inhibitory effect of SPD on α1-adrenoceptor-mediated contractile responses evoked by nerve-released and exogenous noradrenaline in peripheral small- resistance arteries might contribute to its antihypertensive effect  [48].

In addition, SPD has been reported to inhibit Ca^2+^ release and block Ca^2+^ channels. Using aortic strip preparations, a low dose (0.3-100 μM) of SPD inhibited norepinephrine (NE)-induced Ca^2+^ release from intracellular stores [58] whereas a high dose (3-30 mM) blocked voltage-dependent Ca^2+^ channels [50, 52, 58]. SPD also inhibited the elevation of cytosolic free Ca^2+^ induced by high extracellular Ca^2+^ and NE [50]. However, the blocking effect on Ca^2+^ channels by SPD was weaker than that of verapamil (a calcium channel blocker) [77].

Collectively, SPD reverses the contraction of peripheral arteries in cerebral, mesenteric, renal and coronary regions, and results in a decrease of total peripheral resistance, thus leading to its antihypertensive effects [59].

## 6. NEUROPROTECTIVE EFFECTS OF SPD

Morphological and biochemical experiments have demonstrated that SPD possesses neuroprotective effects [66]. The possible pharmacological mechanisms of SPD-induced neuroprotection are complex. First, in a rat four-vessel occlusion model, SPD prevented ischemia-induced inhibition of calcium/calmodulin-dependent protein kinase II (CCDPKII) [66]. The preservation of activity of CCDPKII may be involved in the mechanism of neuronal protection against ischemia [69]. Second, SPD reduced lactate dehydrogenase, an indicator of injury, from neurons following ischemia, suggesting that SPD protects neurons against hypoxia-induced injury, which has been further confirmed by histological studies in striatal neurons [66]. Third, the neuroprotective effects of SPD may be related to its ability to scavenge hydroxyl free radicals. It was reported that SPD and its analogs (i.e., THPBs) decreased the concentration of malondialdehyde brain homogenate and scavenged OH^·^ [38]. Recently, Zhang *et al*. found that SPD increased levels of phospho-AKt, thus keeping neurons viable following exposure to H_2_O_2_ neurotoxicity. These results suggest that SPD can maintain cell survival following exposure to certain neurotoxins or serve as an antiapoptotic factor [80]. Finally, SPD increased the number of both microtubule-associated protein-2-immunoreactive cells and proliferated precursor cell spheres in cultured striatal precursor neurons pretreated with fibroblast growth factor-2. In addition, SPD also promoted the expression of tyrosine hydroxylase in these cells. These results suggest that SPD may regulate neuronal proliferation and promote neuronal differentiation of striatal-derived neural precursor cells [25].

Considering all of its effects, SPD may be utilized as a cytoprotective drug to attenuate ischemia-induced neuronal injury, thereby maintaining neuronal survival, and may even be used to promote neuronal regeneration. These properties of SPD suggest potential therapeutic applications for the treatment of neurodegeneration.

## 7. POTENTIAL APPLICATIONS

### 7.1. SPD for the Treatment of Schizophrenia

Schizophrenia is a chronic, devastating mental disorder. It is characterized by the presence of both negative and positive symptoms. Schizophrenia is the most serious of all psychiatric illnesses and affects about 1% of the world's population, but the etiology of schizophrenia remains obscure. According to recent clinical and experimental studies, the cause of schizophrenia may involve an imbalance of cortical/subcortical DA systems. Specifically, hyperactivity of D2 receptors in subcortical mesolimbic regions, such as the NAc, may produce the positive symptoms of schizophrenia, while hypofunction of D1 receptors in the mPFC is considered to be responsible for the negative symptoms and cognitive impairment [9, 14, 55].

D1 and D2 receptors have important functional interactions (see section 2). However, these interactions were abnormal in over half of the postmortem striata from schizophrenic patients, suggesting an impaired functional linkage between D1 and D2 receptors in schizophrenic patients [57]. Therefore, the restoration of normal, functional linkage between D1 and D2 receptors may be an effective strategy for the treatment of schizophrenia.

Before atypical neuroleptics were discovered, clinical doses of the available antipsychotic drugs primarily blocked D2 receptors [13, 56], but unfortunately they were less effective at PFC D1 receptors. As a result, a large proportion of schizophrenic patients did not show adequate relief from their negative symptoms [1]. However, even with the development of atypical neuroleptics, most of them display only very small D1 agonistic effects on the PFC D1 receptor. Therefore, there is an urgent need to develop an agent that exerts both D1 agonistic and D2 antagonistic effects, which should more effectively restore synergism between D1 and D2 receptors and alleviate both the negative and positive symptoms of schizophrenia. SPD possesses these characteristics and may serve as a novel, promising agent for schizophrenia therapeutics.

Clinical trials are currently testing the potential use of SPD as an antipsychotic drug. Co-administration of SPD and typical antipsychotic drugs (e.g., phenothiazines or butyrophenones) could significantly enhance the effectiveness of drug therapy for mental disorders. In addition, SPD alone has been shown to exhibit a significant therapeutic effect. Furthermore, a double-blind comparative study demonstrated that SPD not only improved the positive symptoms of schizophrenia, but also significantly alleviated the negative symptoms compared to perphenazine, a common drug prescribed for schizophrenic patients [36, 37, 74]. Recently, experiments have also demonstrated that SPD exerts its antipsychotic-like effects in a schizophrenic animal model with few extrapyramidal side effects  [18]. The promising therapeutic effects of SPD probably are the result of its dual D1 agonistic and D2 antagonistic effects, which can potentially reverse hyperactivity of D2 receptors in subcortical mesolimbic regions and restore normal function of D1 receptors in the mPFC, thus restoring normal, functional linkage between D1 and D2 receptors.

### 7.2. SPD for the Treatment of PD

PD, after Alzheimer’s disease, is the second most common progressive neurodegenerative disorder in the United States, affecting about one million people, with more than 50,000 new cases being diagnosed each year. Its prevalence in the U.S. population aged over 50-years old is above one percent, and likely exceeds five percent in individuals aged over 80-years old [72]. As a result, PD is a relatively frequent disorder that occurs in older populations.

The pathogenesis of PD typically is slow-paced but relentlessly progressive. Understanding of the etiopathogenesis of PD is still limited. According to current knowledge, PD develops as a consequence of DAergic neuronal degeneration in the SNc. It appears that several factors (such as oxidative stress, glutamate toxicity, and genetics) contribute to this degenerative process [7, 42], but oxygen-containing free radicals, especially OH^·^, may play a key role [2, 19, 42, 53]. Another important factor is that the unique biochemical features of the SN, such as containing “a high content of oxidizable DA, neuromelanin, polyunsaturated fatty acids, and iron, and relatively low antioxidant complement with high metabolic rate” render the SN, particularly its zona compacta, vulnerable to oxidative damage [42]. As a result, the number of surviving DA neurons decreases to less than 20% in the nigrostriatum following the development of PD.

The most accepted therapeutic strategy for the treatment of PD is essentially DA replacement. L-dopa, the direct precursor of DA, in combination with carbidopa, remains the most effective drug combination. The majority of patients taking these drugs experience benefits initially, but drug effectiveness declines after 2-5 years, evidenced by the emergence of significant adverse effects associated with L-dopa. Long-term L-dopa therapy increases oxidative burden [42], thus worsening oxidative stress, neurodegeneration and the loss of DA neurons. Recently, it has been found that long-term L-dopa therapy results in dyskinesia due to the over-expression of D3 receptors in brain-selective nuclei [41]. Therefore, there is a considerable need for new drugs aimed at relieving the motor symptoms associated with PD by directly depressing D3 receptor expression and protecting surviving DA neurons against, or slowing down the progression of, neuronal degeneration in the SN [24, 54].

SPD may be potentially used as a novel therapeutic strategy for PD. As previously described, SPD not only protects striatal cells against transient cerebral ischemic injury [66], but also scavenges hydroxyl free radicals and maintains neuronal survival following exposure to H_2_O_2_ neurotoxicity [38, 80]. More importantly, SPD possesses D3 receptor antagonism, and it has been co-administered with L-dopa for the treatment of PD [46]. Also, SPD and THPB-18 protect DA neurons against damage induced by 1-methyl-4-phenyl-1,2,3,6-tetrahydropyridine and 1-methyl-4-phenyl-pyridinium (a selective DA neuron toxin) [49]. In addition, SPD may promote proliferation and differentiation of neuronal precursor cells [25]. Furthermore, preliminarily clinical trials have demonstrated that SPD, in combination with a low dose of bromocriptine, can alleviate the symptoms of PD [49]. Thus, SPD may be able to exert symptomatic therapeutic effects and prevent further DA neuron degeneration in PD.

Animal experiments have demonstrated that SPD is well-absorbed in the digestive tract, can be widely distributed in body tissues, and can easily penetrate the blood-brain barrier [85]. SPD may also be suitable for patients with PD and hypertension since it can decrease blood pressure. Taken collectively, SPD is a unique, promising novel drug that potentially can be used for the treatment of PD due to its ability to exert both symptomatic therapy and neuroprotection.

## 8. CONCLUSION

SPD has been shown to possess dual D1 agonistic and D2 antagonistic effects in both the nigrostriatal and mesocorticolimbic DAergic pathways. Thus, SPD potentially reverses hyperactivity of subcortical D2 receptors and restores PFC dysfunctional D1 receptors in patients with schizophrenia, which would result in the recovery of functional linkage between D1 and D2 receptors. SPD not only treats the positive symptoms of schizophrenia, but also alleviates the negative symptoms. In addition, SPD also potentially relieves the motor symptoms of PD due to its D1 agonistic and D3 antagonistic effects. Furthermore, SPD exhibits neuroprotective effects by scavenging hydroxyl free radicals, and consequently, results in the slowing down of neuronal degeneration in the SN of patients with PD. Considering its features, such as being well-absorbed in the digestive tract, can be widely distributed in body tissues, and can easily penetrate the blood-brain barrie  [85], as well as its dual pharmacological effects on D1 and D2 receptors, SPD is a unique, novel and promising drug for the treatment of schizophrenia and/or PD.

## ABBREVIATIONS

SPD: = (-)-stepholidine
PD: = Parkinson’s disease
SN: = Substantia nigra
SNc: = Substantia nigra pars compacta
SNr: = Substantia nigra pars reticulata
DA: = Dopamine
THPBs: = Tetrahydroprotoberberines
CNS: = Central nervous system
mPFC: = Medial prefrontal cortex
VTA: = Ventral tegmental area
NAc: = Nucleus accumbens
6-OHDA: = 6-hydroxy-DA
APO: = Apomorphine
L-dopa: = Levodopa
AC: = Adenylate cyclase
i.v.: = Intravenous
SHRs: = Spontaneously hypertensive rats
NE: = Norepinephrine
CCDPKII: = Calcium/calmodulin-dependent protein kinase II
AD: = Alzheimer’s disease
